# Proximity effect in a two-dimensional electron gas coupled to a thin superconducting layer

**DOI:** 10.3762/bjnano.9.118

**Published:** 2018-04-23

**Authors:** Christopher Reeg, Daniel Loss, Jelena Klinovaja

**Affiliations:** 1 Department of Physics, University of Basel, Klingelbergstrasse 82, CH-4056 Basel, Switzerland

**Keywords:** Majorana fermions, mesoscopic physics, proximity effect, quantum computing, topological superconductivity

## Abstract

There have recently been several experiments studying induced superconductivity in semiconducting two-dimensional electron gases that are strongly coupled to thin superconducting layers, as well as probing possible topological phases supporting Majorana bound states in such setups. We show that a large band shift is induced in the semiconductor by the superconductor in this geometry, thus making it challenging to realize a topological phase. Additionally, we show that while increasing the thickness of the superconducting layer reduces the magnitude of the band shift, it also leads to a more significant renormalization of the semiconducting material parameters and does not reduce the challenge of tuning into a topological phase.

## Introduction

Topological superconductors host zero-energy Majorana bound states at their edges that are highly sought for applications in topological quantum computing [1–3]. The two proposals to realize topological superconductivity that have received the most attention to date involve engineering Majorana bound states in either low-dimensional semiconducting systems [4–23] or in ferromagnetic atomic chains [24–32]. After the first signatures of topological superconductivity were observed [33–37], much of the experimental focus was placed on developing more suitable devices for realizing robust topological superconducting phases. One of the most significant experimental advances of the past few years was the successful epitaxial growth of thin layers of superconducting Al on InAs and InSb nanowires [38–42]. The intimate contact between the semiconductor and superconductor in these devices ensures a hard induced superconducting gap. Recently, this epitaxial growth technique has been applied also to InAs two-dimensional electron gases (2DEGs) [43–47].

The proximity effect has been theoretically studied recently in both strictly one-dimensional (1D) [48] and quasi-1D [49] wires coupled to thin superconducting layers. In both instances, a strong proximity coupling induces a large band shift on the semiconducting wire. This band shift is comparable to the level spacing in the superconductor, 
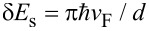
 (which is δ*E*_s_ ~ 400 meV for a superconductor thickness of *d* ~ 10 nm and a Fermi velocity of Al of *v*_F_ ~ 2 × 10^6^ m/s). In both cases, this large band shift makes it very challenging to realize a topological phase when utilizing thin superconducting layers.

In this paper, we extend the works of [48–49] to the 2D limit. We show that the large band shift that plagues the 1D case persists also in two dimensions. First, we show that the self-energy induced in an infinite 2DEG coupled to a superconductor of finite thickness is equivalent to that induced in an infinite wire coupled to a 2D superconductor of finite width (corresponding to the theoretical model of [48]), with the simple replacement of a 1D momentum by the magnitude of a 2D momentum. Analyzing the self-energy, we find that the induced gap in the presence of only Rashba spin–orbit coupling can be made comparable to the bulk gap of the superconductor only if the tunneling energy scale exceeds the large level spacing of the superconducting layer. As in the 1D case, the large tunneling energy scale induces a large band shift on the 2DEG and makes it very challenging to realize a topological phase. We also show that while the band shift can be significantly reduced by increasing the thickness of the superconducting layer, the topological phase is still difficult to realize if the 2DEG/superconductor interface remains very transparent.

## Model of the Proximity Effect

**Figure 1:**
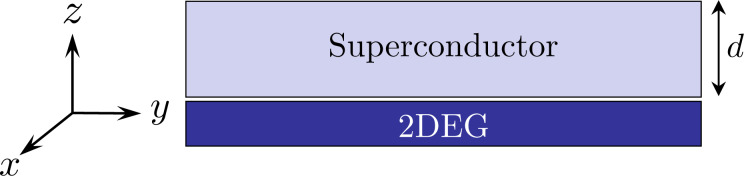
A 2DEG is proximity-coupled to an *s*-wave superconductor with finite thickness *d*. Both systems are taken to be infinite in the *xy*-plane.

The system we consider consists of a 2DEG with strong Rashba spin–orbit interaction (SOI) proximity-coupled to an *s*-wave superconductor of thickness *d*, as shown in Figure 1. The 2DEG-superconductor heterostructure is described by the action

[1]
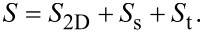


The action of the 2DEG in Nambu space is given by

[2]
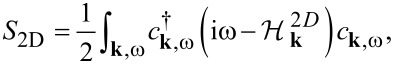


where ω is a Matsubara frequency, **k** = (*k_x_*,*k_y_*) is the momentum,


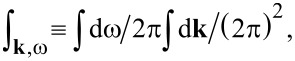


and


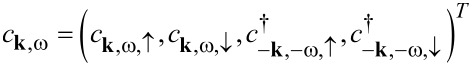


is a spinor of Heisenberg operators describing states in the 2DEG. The Hamiltonian density is

[3]
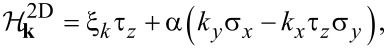


where ξ*_k_* = *k*^2^/2*m*_2D_ − μ_2D_ (*m*_2D_ and μ_2D_ are the effective mass and chemical potential of the 2DEG, respectively, and 
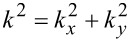
), α is the Rashba SOI constant, and σ*_x,y,z_* (τ*_x,y,z_*) are Pauli matrices acting in spin (Nambu) space. The superconductor is described by the BCS action,

[4]
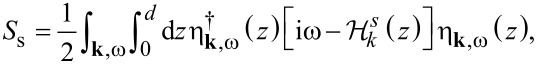


where


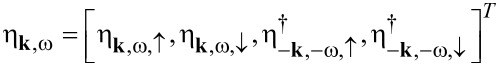


is a spinor of Heisenberg operators describing states in the superconductor and the Hamiltonian density is

[5]
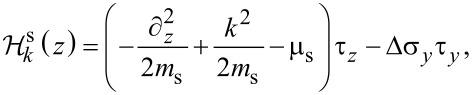


with *m*_s_, μ_s_, and Δ the effective mass, chemical potential, and pairing potential of the superconductor, respectively. Local tunneling at the interface between the two materials is assumed to conserve both spin and momentum,

[6]
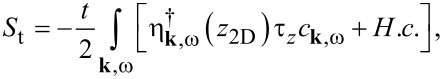


where *t* is the tunneling amplitude. We must take the 2DEG to be located at some finite *z*_2D_ (0 *< z*_2D_ *< d*) due to the breakdown of the tunneling Hamiltonian approach for the case where the 2DEG is located at the boundary of the superconductor. The breakdown of the tunneling Hamiltonian results from our neglect of the thickness of the 2DEG (for related calculations in which the finite thickness is taken into account, see [50–53]). However, as shown in [48], choosing 
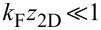
 (where 
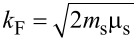
 is the Fermi momentum of the superconductor) yields good agreement with numerical calculations in which there is no issue with placing the 2DEG strictly at the boundary.

In the absence of tunneling, the spectrum of the 2DEG consists of two spin–orbit-split subbands described by

[7]
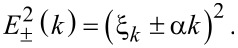


When the finite-size quantization scale of the superconductor greatly exceeds the gap, 
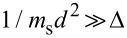
, the first few subbands of the superconductor follow a linearized form given by (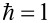)

[8]
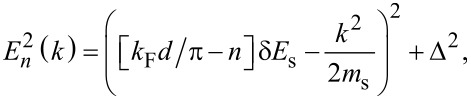


where δ*E*_s_ = π*v*_F_/*d* is the level spacing in the superconductor (*v*_F_ = *k_F_*/*m*_s_ is the Fermi velocity) and 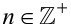. When the thickness of the superconducting layer is much smaller than its coherence length, 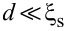 = π*v*_F_/Δ, the level spacing of the layer greatly exceeds its gap, 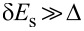.

**Figure 2:**
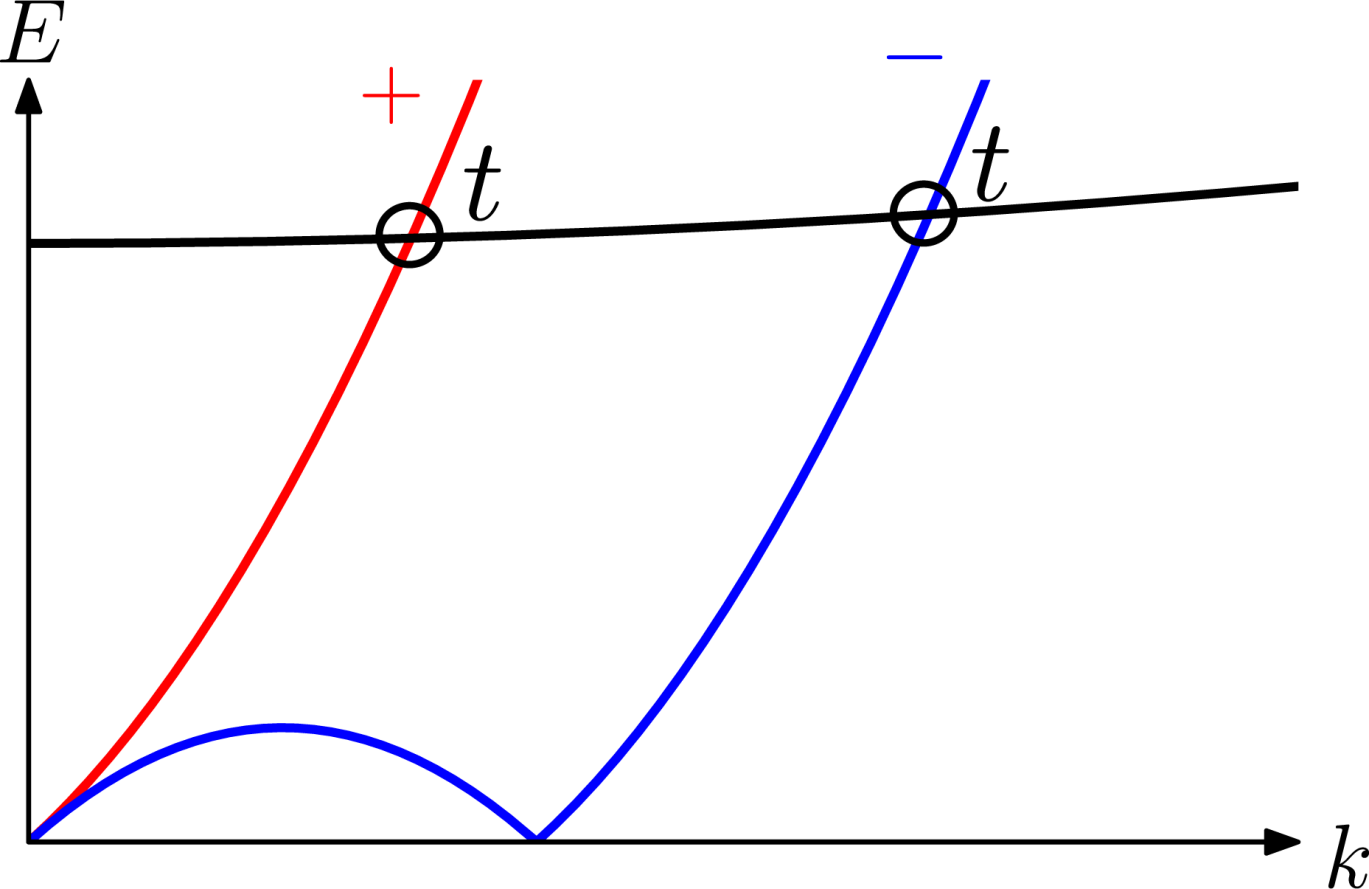
Sketch of Bogoliubov excitation spectra as a function of 
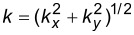
 in the absence of tunneling, assuming 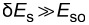 and μ_2D_ = 0. The red and blue curves correspond to ± subbands of the 2DEG (Equation 7), respectively, which result from the spin-splitting Rashba SOI. The black curve corresponds to the lowest-energy subband of the superconductor (Equation 8). A weak tunneling amplitude *t* induces anticrossings in the spectrum where indicated and induces a superconducting gap in the 2DEG at the Fermi momenta (corresponding to those momenta for which *E*_±_(*k*)=0). Due to the large energy mismatch between the superconducting subband and the Fermi points of the 2DEG, the induced gap is very small.

The spectra of the 2DEG and the superconductor are plotted in Figure 2. Provided that 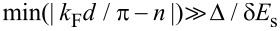, the bands of the 2DEG and superconductor intersect at high energies 
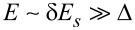
. Since we impose momentum conservation (in addition to energy conservation) in Equation 6, the subbands are coupled only at the intersection points. Thus, a weak tunnel coupling induces anticrossings in the spectrum, as indicated in Figure 2, which leads to a shift in the subbands of the 2DEG. Additionally, the tunnel coupling opens a superconducting gap at the Fermi momenta of the 2DEG; however, due to the intersection points lying at very large energies, the gap opened in the 2DEG is very small. A large gap can only be induced if tunneling is strong enough to overcome the large energy mismatch similar to δ*E*_s_.

To determine the self-energy of the 2DEG induced by the superconductor, we integrate out the superconducting degrees of freedom. After integrating out, the 2DEG can be described by the effective action

[9]
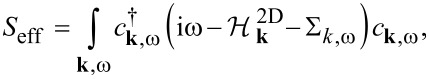


with the self-energy given by

[10]
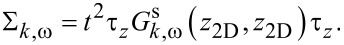


In Equation 10, 
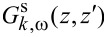
 is the Green’s function of the bare superconductor (in the absence of tunneling), which satisfies

[11]
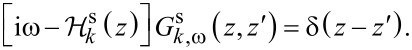


Imposing a vanishing boundary condition at *z* = 0 and *z* = *d*, we find a solution to Equation 11 given by

[12]
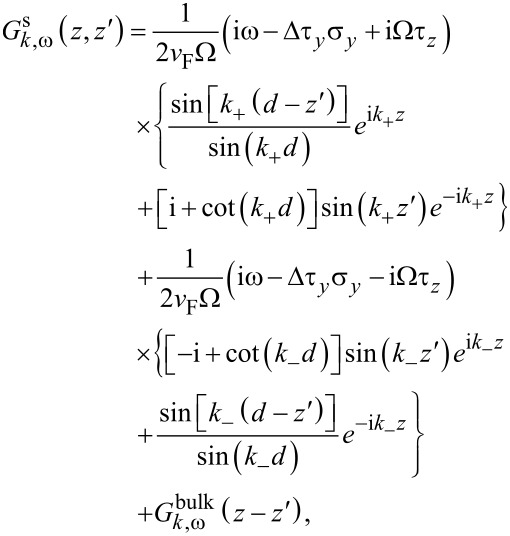


where 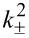 = 2*m*_s_(μ_s_ ± iΩ) − *k*^2^ and Ω^2^ = Δ^2^ + ω^2^ [48,54]. The Green’s function of a bulk superconductor, expressed in real space, is

[13]
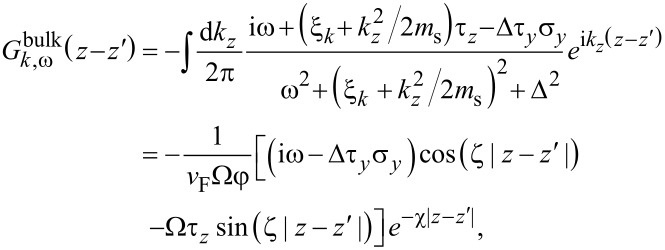


where, in evaluating the integral, we make a semiclassical expansion *k*_±_ = *k*_F_φ ± iΩ/(*v*_F_φ) ≡ ζ ± iχ (valid in the limit 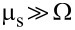) and define a quantity 
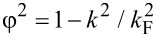
 that parametrizes the trajectories of states in the superconductor. Substituting the Green’s function (Equation 12) into the self-energy (Equation 10), we find

[14]
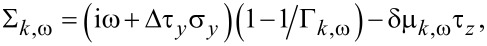


where we define

[15]
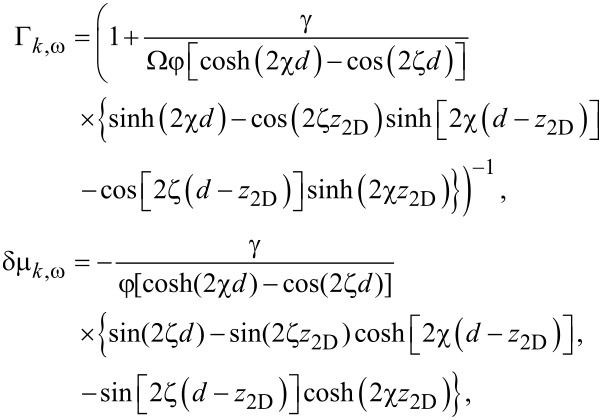


with γ = *t*^2^/*v*_F_, an energy scale determined by the tunneling strength. The quantity Γ*_k,_*_ω_ can be interpreted as an effective quasiparticle weight, as it takes values of 0 *<* Γ *<* 1, and is responsible for inducing superconductivity in the 2DEG, while δμ*_k,_*_ω_ corresponds to a tunneling-induced shift in the effective chemical potential of the 2DEG. Quite surprisingly, the self-energy in Equation 14 and Equation 15 coincides with that of a nanowire coupled to a two-dimensional superconductor with finite width as found in [48], with the simple replacement of a 1D momentum by the magnitude of a 2D momentum.

## Results and Discussion

### Induced gap and band shift

Using the self-energy derived in the previous section, we first calculate the size of the proximity-induced gap in the 2DEG. Once we find an expression for the gap, we estimate the tunneling strength needed in order for the gap in the 2DEG to be comparable to that in the superconductor. We then add a Zeeman term to the Hamiltonian of the 2DEG and estimate the Zeeman energy needed to reach the topological phase in such a setup.

It is convenient to work in the chiral basis in which the normal Green’s function of the 2DEG is diagonal. To this end, we introduce a unitary transformation

[16]
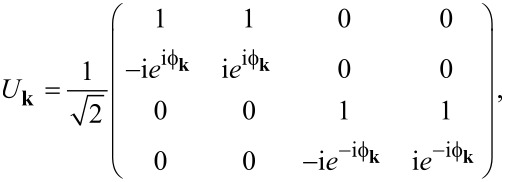


with 
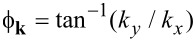
, which can be used to convert between the spin (σ) and chiral (λ) bases, 
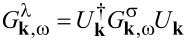
. The Green’s function in the spin basis is given by 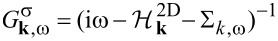. Rotating to the chiral basis, we find a Green’s function given by

[17]
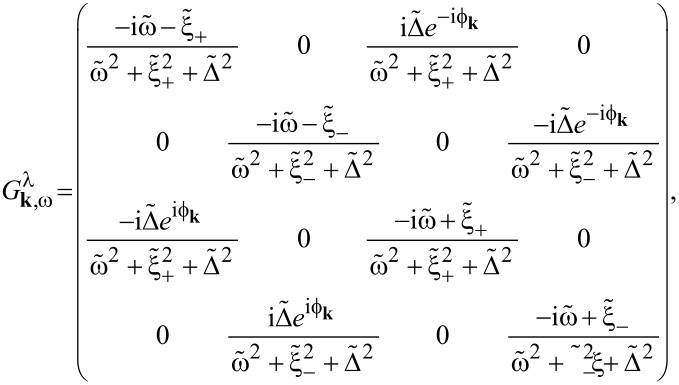


where 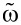 = ω/Γ*_k,_*_ω_, 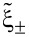 = ξ*_k_* − δμ*_k,_*_ω_ ± α*k* and 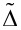 = Δ(1/Γ*_k,_*_ω_ − 1). The spin–singlet pairing induced by the superconductor appears as intraband chiral *p*-wave pairing (of the form *p_x_* ± i*p_y_*) when expressed in the chiral basis.

Before continuing, let us simplify the parameters Γ*_k,_*_ω_ and δμ*_k,_*_ω_. We will focus on the limit where the thickness of the superconducting layer is much smaller than its coherence length, 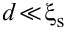 (equivalently, 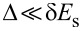), and where the normal layer is located close to the edge of the superconductor, 
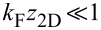
. Because of the large Fermi surface mismatch between the 2DEG and superconductor, we must have 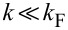 (or, equivalently, φ ≈ 1); in the following, we neglect the momentum dependence by setting φ = 1 (which is justified as long as we only consider momenta 
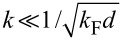
). In the limit 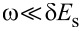, the parameters simplify to

[18]
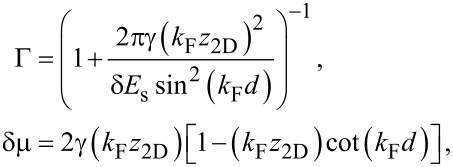


where we drop the subscript (*k*,ω) because both Γ and δμ are now independent of frequency and momentum. In expanding Equation 15 to arrive at Equation 18, we assumed that 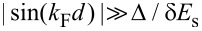 (therefore, these expressions break down when *k*_F_*d*/π→*n*, with 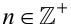).

The spectrum of the proximitized 2DEG is determined by the poles of the retarded Green’s function. After analytic continuation iω→*E* + i0^+^, we find two branches of the spectrum from Equation 17 given by

[19]
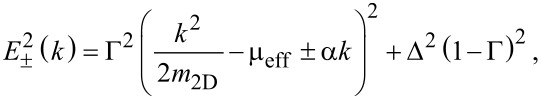


where μ_eff_ = μ_2D_ + δμ is an effective chemical potential of the 2DEG. The spectrum describes an *s*-wave superconductor with Rashba-split bands and an excitation gap

[20]
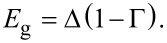


We see that the size of the excitation gap is determined by the parameter Γ. When 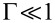, the full bulk gap of the superconductor is induced in the 2DEG, while for 
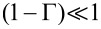
, a very small gap is induced. In order to have an induced gap comparable (but not equal) to the bulk gap, we require that neither 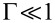 nor 
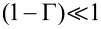
 is satisfied. However, to realize this situation requires a tunneling strength

[21]
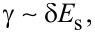


where we have assumed that (*k*_F_*z*_2D_)^2^/sin^2^(*k*_F_*d*) ~ 1. If the tunneling strength is chosen as in Equation 21, the band shift measured at *k* = 0, *E*_±_(0), is

[22]
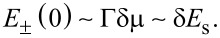


Therefore, the scale of the band shift is also set by the level spacing in the thin superconducting layer. We note that while the quantity δμ is bounded only by the chemical potential of the superconductor μ_s_ (as the tunneling Hamiltonian approach itself should break down for γ ~ μ_s_), the band shift saturates to *E*_±_(0) ~ δ*E*_s_ in the limit 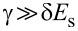 (where 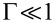).

**Figure 3:**
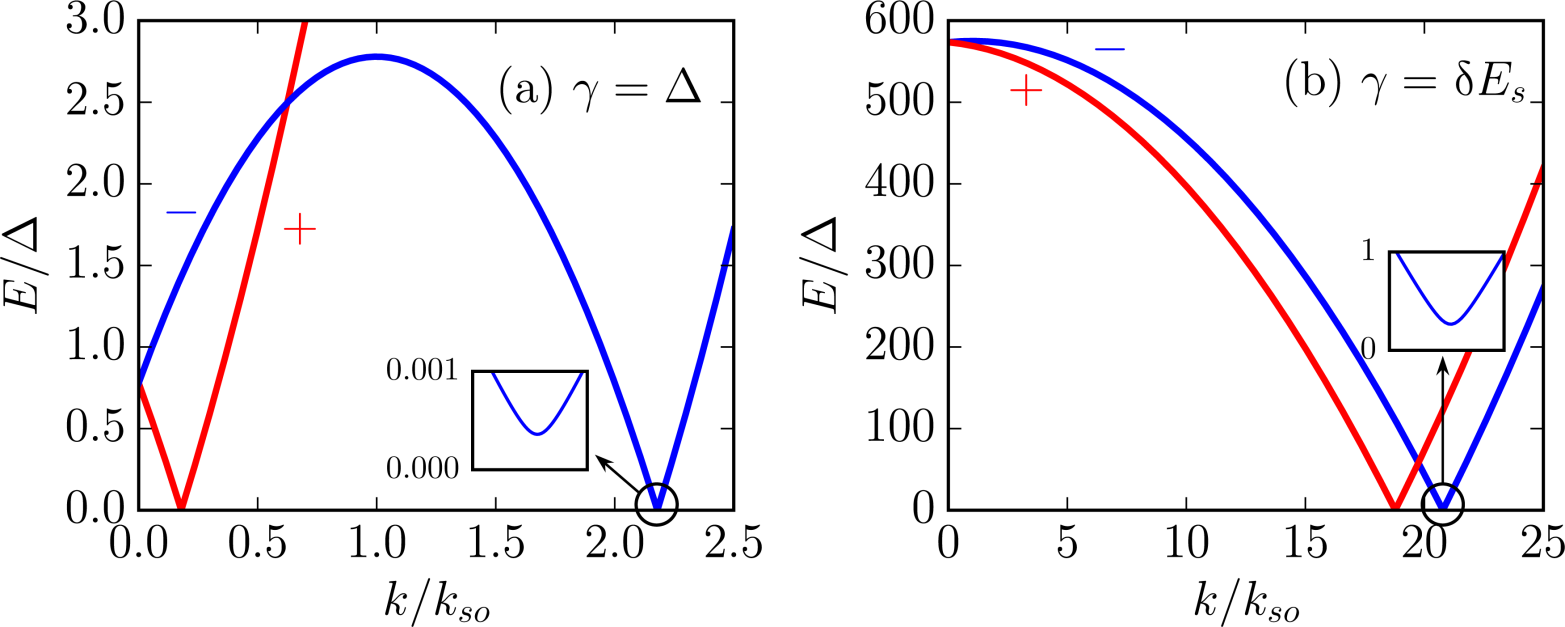
Spectrum of a 2DEG coupled to a thin superconducting layer (see Equation 19) for (a) γ = Δ (corresponding to Γ = 0.9996 and δμ = 0.78Δ) and (b) γ = δ*E*_s_ (corresponding to Γ = 0.735 and δμ = 780Δ). When tunneling is weak (as in panel a), the band shift is rather small but the induced gap is negligible. If tunneling is strong enough to open a sizable gap (as in panel b), the band shift is very large (note that the band shift is given by *E*_±_(0) ~ Γδμ rather than δμ). In both plots, *E*_so_ = 2Δ, δ*E*_s_ = 1000Δ, μ_2D_ = 0, *k*_F_d/π = 48.75, and *k*_F_*z*_2D_ = 0.3. Here *k*_so_ = *m*α is the spin–orbit momentum. Note that although, in the insets, we show only the induced gap on the "−"-subband, there is an equally large gap induced on the "+"-subband.

We plot the spectrum of the 2DEG (see Equation 19) in Figure 3. In the weak-coupling limit (Figure 3a), there is a rather small band shift but a negligible superconducting gap is opened in the 2DEG. In the strong-coupling limit (Figure 3b), we show that while a larger gap is induced, the band shift is very large.

### Topological transition

We now add a Zeeman splitting Δ_Z_ to the Hamiltonian of the 2DEG such that

[23]
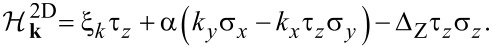


Such a Zeeman splitting can arise due to the application of an out-of-plane magnetic field [4–5] (though orbital effects are not incorporated here) or due to the proximity of a magnetic insulator [8]. Also, it is possible to apply an in-plane magnetic field (to avoid unwanted orbital effects) to reach the topological phase if the 2DEG has a finite Dresselhaus SOI, as shown in [9]. An in-plane magnetic field in the presence of only Rashba SOI is not sufficient to reach the topological phase because it does not open a gap in the Rashba spectrum. The spectrum in the presence of the Zeeman splitting, which again is determined by poles in the retarded Green’s function 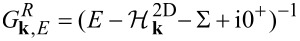, is given by

[24]
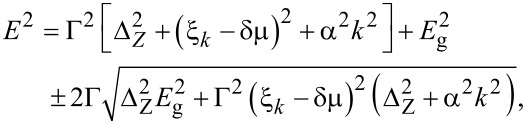


where we have used *E*_g_ = Δ(1 − Γ) as in Equation 20. Therefore, we find a gap-closing topological transition at *k* = 0 for the critical Zeeman splitting

[25]
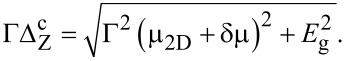


In the case of a very large band shift, 
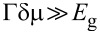
 and 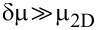, the topological transition is given by 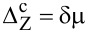 [note that Γ drops out of Equation 25 in this limit].

We now provide an estimate of the Zeeman splitting at which we expect the *k* = 0 gap-closing transition to occur experimentally in an Al/InAs 2DEG heterostructure. Given the thickness of the superconducting Al layer of *d* = 10 nm [44], we estimate a level spacing of δ*E*_s_ = 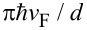 = 413 meV (taking *v*_F_ = 2 × 10^6^ m/s). Therefore, if a sizable gap is induced in the 2DEG, as observed experimentally, typical values for the band shift are of the same order of magnitude as the level spacing, Γδμ ~ 400 meV. Then, provided that the chemical potential cannot be controlled over such a large scale by external gates, the critical Zeeman splitting needed to reach the topological phase is 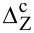 = δμ ~ 400 meV. Such a large Zeeman splitting cannot be achieved in the 2DEG without destroying superconductivity in the thin layer. We also note the possibility that, by coincidence, the band shift vanishes (or becomes small); from Equation 18, we see that δμ = 0 if *k*_F_*d* = cot^−1^(1/*k*_F_*z*_2D_) + *n*π (for 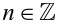). In this special case, which requires the thickness of the superconducting layer to be finely tuned on the scale of its Fermi wavelength, there is no band shift to prevent one from tuning into a topological phase. However, for most devices, the large band shift makes it very challenging to realize a topological phase.

### Increasing thickness of superconducting layer

The self-energy appearing most frequently in the literature to describe proximitized nanowires and 2DEGs [55–60], which also has been used often in interpreting experimental results [40,42], is that induced by a bulk superconductor,

[26]
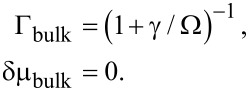


**Figure 4:**
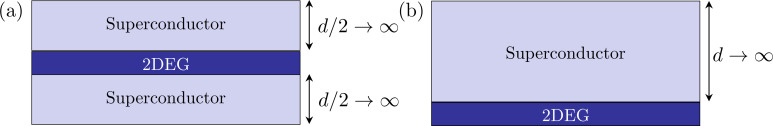
(a) Evaluating the self-energy with the Green’s function of a bulk superconductor (see Equation 26) corresponds to a 2DEG embedded within an infinitely large superconductor. (b) Evaluating the self-energy with the Green’s function of a semi-infinite superconductor (see Equation 27) corresponds to a 2DEG placed on the surface of an infinitely large superconductor.

Equation 26 can be obtained by setting *z*_2D_ = *d*/2 and taking the limit *d*→∞ in Equation 15 (or, as it is usually done, by substituting the bulk Green’s function in Equation 13 when evaluating the self-energy in Equation 10). Hence, this self-energy describes a 2DEG embedded within a bulk superconductor, as shown in Figure 4a. To describe the case where a 2DEG is placed at the surface of a bulk superconductor (as shown in Figure 4b), the limit *d*→∞ should be taken in Equation 15 while keeping *z*_2D_ finite (or, equivalently, substituting the Green’s function of a semi-infinite (SI) superconductor when evaluating the self-energy in Equation 10). For this case, we obtain

[27]
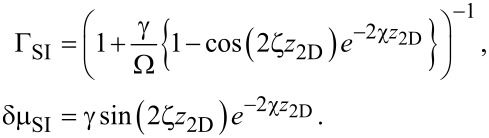


The most notable difference is the presence of a nonzero band shift in the semi-infinite case. However, this band shift is significantly reduced compared to the case of a thin superconducting layer, as it saturates to *E*_±_(0) ~ Γ_SI_δμ_SI_ ~ Δ in the limit 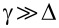.

Although it may seem that a topological phase can be much more easily realized by simply increasing the thickness of the superconducting layer in order to reduce the band shift induced on the 2DEG, this is not the case. Crucially, both the bulk and the semi-infinite self-energies give the ratio γ/Δ as the relevant parameter determining whether the system is in the weak-coupling [
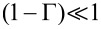
, or equivalently 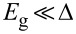] or strong-coupling [(1 − Γ) ~ 1, or equivalently *E*_g_ ~ Δ] limit. This is in stark contrast to the limit of a thin superconducting layer, where a tunneling energy 
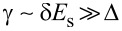
 is required to open a gap *E*_g_ ~ Δ in the 2DEG. Therefore, because the tunneling energy γ is a property of the interface and should not be expected to change as the thickness of the superconducting layer is increased, this energy is fixed to γ ~ δ*E*_s_ provided that the interface is transparent enough to induce a gap in the thin-layer limit (as seen in the experiments). If the thickness of the superconductor is increased, such that 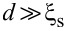, the system will be deep within the strong-coupling limit; from Equation 26 and Equation 27, we find 
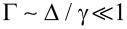
. The critical Zeeman splitting needed to induce a topological phase (see Equation 25) is therefore given by 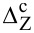 ~ Δ/Γ ~ γ ~ 400 meV. We note that in the case of a thin superconducting layer, the topological transition is pushed to large Zeeman splitting by very large δμ, which could possibly be compensated for if the chemical potential μ_2D_ has a large range of tunability. In the case of a bulk system, the topological transition is pushed to large Zeeman splitting by very small Γ, which cannot be affected by tuning μ_2D_. Hence, even if the thickness *d* of the superconducting layer is made infinite, the topological phase transition is determined by the interfacial tunneling energy. In order to induce a topological phase more reliably, a much weaker coupling between a 2DEG and a bulk superconductor (such that γ ≤ Δ) should be sought. We also note that this result applies to the 1D model considered in [48] as well.)

## Conclusion

We have studied the proximity effect in a two-dimensional electron gas (2DEG) strongly coupled to a thin superconducting layer, showing that the detrimental band shift shown in [48–49] to dominate the proximity effect in wires is also crucial in 2DEGs. In order to induce a sizable gap in the 2DEG, the tunneling energy scale must overcome the large level spacing within the superconductor. However, introducing such a large energy scale to the semiconductor induces a large band shift that makes it challenging to realize a topological phase. This challenge cannot be alleviated by simply increasing the thickness of the superconducting layer but requires a significant weakening of the proximity coupling afforded by the epitaxial interface.
